# Spermatic Microbiome Characteristics in Infertile Patients: Impact on Sperm Count, Mobility, and Morphology

**DOI:** 10.3390/jcm11061505

**Published:** 2022-03-09

**Authors:** Clémence Gachet, Manon Prat, Christophe Burucoa, Philippe Grivard, Maxime Pichon

**Affiliations:** 1 Laboratory of Andrology and Medically Assisted Reproduction Laboratory, Centre Hospitalier Universitaire de Poitiers, 86021 Poitiers, France; clemence.gachet@chu-poitiers.fr (C.G.); philippe.grivard@chu-poitiers.fr (P.G.); 2 Bacteriology Laboratory, Infectious Agents Department, Centre Hospitalier Universitaire de Poitiers, 86021 Poitiers, France; manon.prat@chu-poitiers.fr (M.P.); christophe.burucoa@chu-poitiers.fr (C.B.); 3 INSERM Institut National de la Santé et de la Recherche Médicale U1070 Pharmacology of Antimicrobial Agents and Resistance, University of Poitiers, 86073 Poitiers, France

**Keywords:** microbiome, infertility, spermatozoa, high-throughput sequencing, omics

## Abstract

Through sperm alteration, semen microbiota tend to be recognized as a cause of infertility, but due to the limited number of studies focusing on this ecological niche, this hypothesis remains controversial. This study aimed to characterize and compare the bacterial communities of sperm samples from patients undergoing couple infertility treatment at the time of diagnosis. The study was prospective (September 2019 to March 2020), monocentric, and focused on alterations of spermatic parameters: count, motility, and morphology. After the amplification of the 16S rDNA (V1 to V3), libraries (*n* = 91, including 53 patients with abnormalities) were sequenced using the MiSeq platform (Illumina). After quality control processing using a homemade pipeline (QIIME2 modules), the main genera were: *Prevotella*, *Finegoldia*, *Pseudomonas*, *Peptinophilus*, *Streptococcus*, *Anaerococcus* and *Corynebacterium*. Restricted diversity was observed in samples from patients with abnormal sperm morphology (α-diversity, *p* < 0.05), whereas diversity increased in patients with an abnormal sperm count (β-diversity, *p* < 0.05). The enrichment of the genus *Prevotella* and *Haemophilus* was observed in negative sperm culture samples and samples with abnormal counts, respectively (*p* < 0.05). Microbiota differed in their composition according to sperm parameters. Finally, this work highlights the need for the optimization of the management of couples undergoing infertility treatment, possibly by modulating the genital microbiome.

## 1. Introduction

The World Health Organization considers infertility to be an important public health problem, as its frequency generally ranges from 15 to 25% [1]. The male contribution to a couple’s infertility tends to appear in more than one half of consultations, even if the precise etiology of these alterations remains undefined in 30 to 50% of cases [2,3]. In most cases, decreased fertility in men is associated with altered sperm parameters, including count, motility, and morphology [4]. Nevertheless, several factors could explain these alterations, including the presence of bacteria in the semen, which are commonly named “bacteriospermia”. A number of in vivo studies have shown that an alteration of sperm parameters frequently occurs in patients with a positive sperm culture [5,6,7,8,9]. However, the true impact of bacterial infections on male fertility remains controversial. Indeed, the negative impact of bacteria on semen parameters has been called into question over recent years, particularly due to the characterization of urogenital microbiomes. Far from being deleterious, the presence of microorganisms in an ecosystem may contribute to physiological balance [10].

Over a long period of time, the estimation of this biodiversity was biased by culture methods that precluded the growth and identification of certain bacteria. Due to the democratization of high-throughput sequencing techniques and the increasing power of bioinformatics, the characterization of different human microbiomes has been at the center of various projects aimed at exploring the relationship between microbiota profile and health [11]. To date, observations and studies have mainly focused on the digestive and respiratory microbiomes, which are considered to be true organs on account of their physiological effects [12]. Similarly, promising studies of vaginal and endometrial microbiomes have been conducted on female genital tract samples in humans [13]. On the other hand, the implication of semen microbiota on male fertility has been minimally investigated.

This prospective clinical study aimed: (i) to determine the composition and structure of bacterial communities in sperm samples taken from infertile couples at the time of diagnosis; (ii) to identify specific taxa associated with male fertility alterations. As the key examination of male infertility is the spermogram, three major parameters were considered: sperm count, motility, and morphology.

## 2. Methods

### 2.1. Selected Samples

Patients were prospectively recruited at Poitiers University Hospital during consultations at the Fertility unit between September 2019 and March 2020.

Patients were included if they fulfilled all the following criteria: (i) they were followed in a medically assisted reproduction program; (ii) they were aged from 18 to 50 years; and (iii) they produced an ejaculate volume greater than 1.5 mL. They were excluded if they were patients who presented at least one of the following criteria: (i) there was delay in abstinence that did not comply with WHO recommendations (2 to 8 days) [14] (WHO guidelines 2010); or (ii) there was a known/suspected cause responsible for sperm abnormalities (i.e., history of genetic or chromosomal disease, history of cryptorchidism during childhood, history of varicocele, testicular surgery, history of testicular cancer, chemotherapy, radiotherapy). For the purpose of this study, no azoospermic patients were included, as no motility and morphology parameters could be considered.

In accordance with the 2010 WHO Guidelines, sperm samples were processed for routine semen assessment at the laboratory of andrology [14]. Semen samples were collected after appropriate sexual abstinence (two to eight days) by masturbation after hand and perineal (including penis) disinfection (with sodium hypochlorite) and then examined after 30 min of liquefaction. Thereafter, the samples were manually evaluated for volume determination, pH, and then, using optical microscopy evaluation, validated on sperm parameters (sperm count, mobility, and morphology). For sperm morphology, smears were stained using the Papanicolaou method (kit Spermoscan, RAL Diagnostics, Martillac, France) and examined under a microscope (×100 magnification) using Kruger classification before being split into different parts and sent to different laboratories [15]. Samples were split into two different aliquots, and a dedicated part of the collected volume (800 µL) was sent to the Infectious Agents Department (CHU Poitiers, France) for spermoculture (using 250 μL) and STI-specific PCR (using 300 µL) (Allplex STI-Essential Assay, Seegene, Seoul, South Korea). The sperm aliquot dedicated to conventional microbiology was plated on blood agar (COS, CHOC, ANC, BioMérieux, Marcy-l’Etoile, France) before incubation for two to five days under 5% CO_2_ ambiance.

In addition to the volume used for routine diagnosis (as previously described), 250 μL of semen samples was mixed with RNAlater (1:2 ratio) in a LowBinding tube (Eppendorf, Hambourg, Germany) before freezing and conservation at −70 °C until analysis.

### 2.2. Sample Preparation

#### 2.2.1. Nucleic Acid Extraction

DNA extraction was performed using a QIAmp^®^ PowerFecal^®^ Pro DNA kit (Qiagen, Venlo, The Netherlands) with bead-beating protocol and enzymatic lysis, according to the manufacturer’s recommendations. Nucleic acids were eluted in 50 µL of Low TE buffer (Eurobio, Les Ulis, France) and then quantified using the dsDNA High Sensitivity assay (Invitrogen, Life Technologies, Carlsbad, CA, USA) on a Qubit 4.0 fluorometer (Invitrogen) before dilution and the initiation of the library production process. A negative control (no-template control) was added to the process and thereafter considered as other clinical samples. A positive control (ZymoBIOMICS Microbial Community Standard) was applied for each experiment to validate the analytical and bioinformatic process.

#### 2.2.2. Library Preparation and Sequencing

Libraries were then produced following fusion PCR targeting V1–V3 16s rDNA regions, as described previously [16]. Briefly, PCR reactions were carried out at a total volume of 25 μL for each sample, containing final concentrations of 1X KAPA HiFi HotStart ReadyMix (Roche, Bâle, Switzerland), 1μM 16S-V1 (27F) forward primer, 1 μM 16S-V3 (536R) reverse primer, and 10 ng of template DNA per reaction. As with other publications focusing on the sperm microbiome [17,18], PCR was carried out using the following cycling conditions: 95 °C for 3 min; 35 cycles of 95 °C for 30 sec, 55 °C for 30 sec, and 72 °C for 30 sec; 72 °C for 5 min and kept at 4 °C until visualization on a 1.5% agarose gel. Validated PCR products were then purified using NucleoMag NGS Clean-up and Size Select beads (Macherey-Nagel, Hoerdt, France) before quantification using the Qubit 1x ds DNA HS Assay Kit (Invitrogen) on the Qubit 4.0 Fluorometer (Invitrogen). Indexing was performed using the Nextera^®^ XT Index Kit V2 Set D (Illumina, CA, USA) according to the manufacturer’s recommendations for fusion PCR. After final quantification using the Qubit 4.0 Fluorometer, all libraries were pooled in equimolar amounts for 96-sample total libraries. After a final dilution to 15 pM, denatured and 5% PhiX-spiked 15 pM pool was sequenced using a MiSeq V3 paired-end kit. 

### 2.3. Bioinformatic Analyses

#### 2.3.1. Quality Filtering and Diversity Analysis

Microbiome bioinformatics were performed with QIIME 2 2018.8 [19]. Raw sequence data were demultiplexed, trimmed at a length of 280 bp and quality filtered (for an expected error rate of less than 0.1%) using the q2-demux plugin, followed by denoising with DADA2 [20]. All amplicon sequence variants (ASVs) were aligned with mafft and used to construct phylogeny with fasttree2 against the Silva132 99% OTUs sequences [21,22,23]. After rarefaction (subsampling without replacement to 7500 sequences per sample), alpha diversity (intra-host diversity) metrics (observed, Chao1′s, Shannon’s indexes, and Faith’s phylogenetic diversity [24]), and beta diversity (inter-host diversity within a group) metrics (weighted UniFrac [25], unweighted UniFrac [26], and Bray–Curtis dissimilarity) were estimated. ANCOM and linear discriminant analysis effect size (LefSe) were used to identify differentially abundant genera among the groups [27]. Although statistically limited, all biological semen characteristics were categorized for analysis because of inter-operator variability in assessing semen parameters to limit information biases.

#### 2.3.2. Relative Abundance Graphs

Taxonomic relative abundance graphs were prepared, dividing microbiome populations into their respective taxonomic levels (phylum, genus, species). Data were analyzed using Student’s *t*-test for statistical significance and reported as follows: *p* ≤ 0.05 (*), ≤0.01 (**), and ≤0.001 (***). Figures were produced using the Qiime2View 2018.8 website. Raw data are available under the Sequence Read Archive BioProject PRJNA647447 (SRR12276169 to SRR12276257).

## 3. Results

### 3.1. Study Population

**Figure 1:**
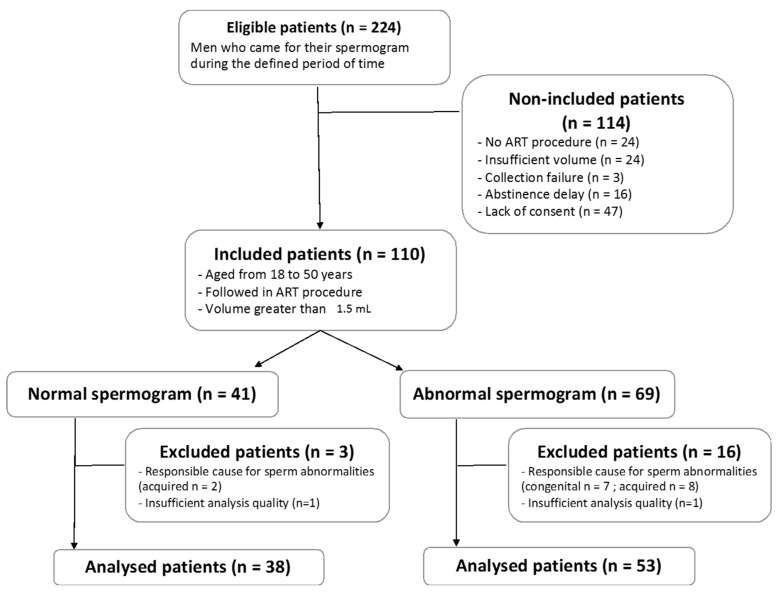
Flow chart of the study population. ART = assisted reproduction technique.

During the inclusion period, 224 patients underwent spermograms. After the application of the selection criteria, 91 patients (91/224: 40.6%) were included for analysis (Figure 1).

In the analyzed cohort, 38 patients (38/91; 41.8%) had a spermogram without abnormality, while 53 patients (53/91; 58.2%) presented with at least one observed abnormality (sperm count, motility, or/and morphology). Demographic and clinical characteristics are summarized thereafter (Table 1). Note that positive controls were valid, and that negative controls did not produce sufficient data after applying filtering parameters described therebefore, validating the following results.

### 3.2. Alteration of the Sperm Microbiome Diversities Is Associated with Sperm Abnormalities

The most abundant bacterial genera in sperm samples (regardless of the results of their semen analysis) included *Firmicutes*; *Bacteroidetes*; *Proteobacteria*, and *Actinobacteria*. Representative members belonged to the *Peptinophilus*, *Streptococcus*, *Finegoldia*, and *Anaerococcus* (for *Firmicutes*); *Prevotella* (for *Bacteroidetes*); *Pseudomonas* and *Serratia* (for *Proteobacteriae*); and *Corynebacterium* sp. (for *Actinobacteriae*) species.

**Figure 2:**
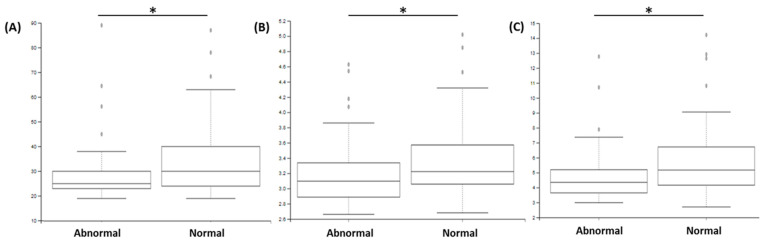
Comparison of alpha diversities, determined with different indexes ((**A**) Chao1; (**B**) Shannon; (**C**) FaithPD) according to the “morphology” parameter. The comparison between groups with abnormal vs. normal morphology demonstrated significant difference (* *p* < 0.05).

Analyses of alpha diversity (based on a comparison of Chao1, Shannon and FaithPD indices; Figure 2.) statistically demonstrated that samples from patients with abnormal sperm morphology presented lower alpha diversity compared to those of samples from patients with normal morphology (*p* < 0.05). On the contrary, no statistical difference could be observed regarding the motility and sperm count parameters (*p* > 0.05). Moreover, samples with negative spermocultures presented statistically lower alpha diversity compared to that of patient samples with positive spermocultures (*p* < 0.05).

**Table 1:** Patients’ baseline characteristics according to sperm parameters: motility, sperm count, and morphology. No statistical association could be observed between sperm parameters and clinical characteristics in the present cohort (*p* > 0.05).

Clinical Characteristic	Motility		Sperm Count		Morphology	
	Motility < 32% (*n* = 29; 31.8%)	Motility ≥ 32% (*n* = 62; 68.2%)	Sperm Count ≤ 39 Millions per Ejaculate (*n* = 20; 22.0%)	Sperm Count > 39 Millions per Ejaculate (*n* = 71; 78%)	Normal Morphology < 4% (*n* = 38; 41.8%)	Normal Morphology ≥ 4% (*n* = 53; 58.2%)
**Age (in years)** **(med; [IQR])**	32 [31–35]	34.5 [30–37.8]	33 [30.5–37.8]	33 [30–37]	33.5 [31–36.8]	33 [30–38]
**Duration of** **sexual abstinence in days (med; [IQR])**	4 [3–5]	4 [3–5.8]	4 [2.8–5]	4 [3–6]	4 [3–5]	4 [3–6]
**Ejaculate volume (in mL) (med; [IQR])**	3.2 [2.5–4.3]	3.9 [2.8–4.7]	3.4 [2.5–3.9]	4 [2.9–4.8]	3.3 [2.6–4.3]	3.9 [2.8–4.7]
**Tobacco consumption** **(ongoing/stopped/never smoked)**	7/5/15	21/14/19	5/3/8	23/16/26	13/5/15	15/14/19
**Spermoculture results** **(% of positive results)**	5 (17.2%)	17 (27.4%)	2 (10%)	20 (28.2%)	7 (18.4%)	15 (28.3%)

**Figure 3:**
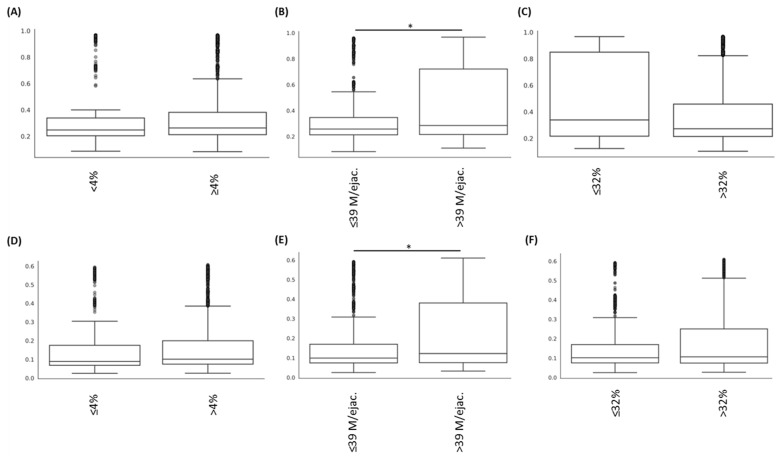
Comparison of beta diversities, determined with different indexes ((**A**–**C**): Bray–Curtis indexes and (**D**–**F**): Unifrac weighted indexes) according to the “Morphology” (**A**,**D**); “sperm count” (**B**,**E**), and “motility” (**C**,**F**) parameters. The comparison between groups with abnormal vs. normal sperm count demonstrated significant difference (* *p* < 0.05).

Analyses of beta diversity (based on Bray–Curtis and Unifrac weighted indices; Table 2 and Figure 3) statistically demonstrated that samples from patients with abnormal sperm counts showed greater beta diversity than samples from patients with normal sperm counts (*p* < 0.05). No difference could be observed regarding the motility and morphology parameters (*p* > 0.05). Analyses of beta diversity (based on Jaccard and Unifrac unweighted indices), demonstrated that samples from patients with negative spermocultures had lower diversity than samples from patients with positive spermocultures (pseudoF: 1.86; *p* < 0.05 and pseudoF: 2.21; *p* < 0.05).

### 3.3. Sperm Abnormalities Are Associated with Modification in Specific Bacteria

ANCOM and linear discriminant analysis effect size (LefSe) approaches demonstrated that *Mobilincus* and *Finegoldia* were significantly enriched in the group that included normal semen morphology patients (*p* < 0.05). Similarly, *Cutibacterium* and *Gordonia* were significantly enriched in the group including patients with normal semen sperm count, while *Haemophilus* was significantly enriched in the group with abnormal semen sperm count (*p* < 0.05). Finally, *Prevotella*, *Peptinophilus*, *Murdochiella*, *Dialister*, and *Haemophilus* genus bacteria were significantly enriched in the group with negative spermoculture (*p* < 0.05).

## 4. Discussion

This study is one of the first French cohorts to focus on the semen microbiota of patients without documented pathology affecting spermatic parameters. While most of the bacteria have proven their pathogenicity on spermatozoa, leading to a major impact on fertility, the presence of bacteriospermia without sperm alterations suggests an absence of strict pathogenicity. In order to standardize the studied cohort, the present study was restricted to patients with abstinence of two to eight days, without a known/suspected cause responsible for sperm abnormalities. This study is original by design, focused only on groups of infertile patients (with or without sperm abnormalities), without comparison of fertile “healthy” patients, who are extensively described in the literature, and considering only semen parameters such as biological outcome. The latter characteristics facilitate focus on the male component of the infertility, without consideration for the female part of this function (deeply studied in the literature, as reviewed by Paula Punzón-Jiménez et al. [28]). While this feature deviates from the inclusion criteria of Hou et al., it could be considered to be quasi-similar to that of Weng et al. and Baud et al. [29,30] (Table 3). The design of the present study has not included comparison to urine microbiota for two reasons. First, this study was considered to be ethically easily acceptable by the authors’ institution due to the very low intervention compared to usual clinical management (which would not include urine sampling in infertile patients). Second, a previous study by Lundy et al. demonstrated that the two microbiomes are similar, bringing no new information regarding the clinical hypotheses [31]. Nevertheless, a limitation must be noted: by design, the present study included all patients entering the assisted reproductive technology process, without excluding those who reported actively/passively smoking. It is conceivable that the latter could have an impact on the composition of the sperm microbiome, even in the absence of specifically targeted studies on this issue (which need to be conducted).

Microbiome studies have introduced the notion of “dysbiosis”, which could be defined as a “possible imbalance from a normal state” [32]. This imbalance could be reflected by a change in composition or abundance of the whole microbial community or of specific microorganisms. The latter limit the validity of Koch’s postulate (indicating that a pathogen leads to a distinct disease), but rather highlight that a comprehensive and complex interpretation (associated with deep understanding) of the interactions between the host, microbial communities, and invasive pathogens is needed. In contrast to ecological niches such as the respiratory or gastrointestinal tracts, a very limited number of studies applying ultra-deep sequencing have focused on the spermatic microbiome, justifying the limited description of the bacterial communities present in the sperm and impacting its functioning. So far, as shown by Carmen Lok Tung Ho et al., the disruption of the sperm microbiome is associated with increased oxidative stress. In seminal plasma, these substances have been clearly associated with sperm abnormalities, such as increased sperm DNA fragmentation and possible alterations in sperm morphology [33].

Herein, bacterial genera *Prevotella*, *Finegoldia*, *Pseudomonas*, *Peptinophilus*, *Streptococcus*, *Anaerococcus*, and *Corynebacterium* were observed as major components of the sperm microbiome. These were initially described in 2008 by Kiessling et al., who performed the first sequencing of 16S rDNA in the semen of patients undergoing infertility or pre-vasectomy [34]. In addition to the genera of the present study, they observed two other major bacterial genera: *Staphylococcus* and *Lactobacillus*. In 2013, Hou et al., using pyrosequencing of 16S RNA gene (V1-V2 regions), described six types of spermatic bacterial communities, called “spermotypes”, dominated by *Streptococcus*, *Corynebacterium*, *Finegoldia*, *Prevotella*, *Peptinophilus*, or *Anaerococcus* [35] (Table 2). In 2014, Weng et al. (using V4 regions of 16S RNA gene) completed this list by highlighting three types of spermatic bacterial communities dominated by *Lactobacillus*, *Prevotella*, or *Pseudomonas*. For the first time, they associated *Prevotella* and *Pseudomonas* with a significant alteration in sperm parameters (20% and 12.5% of normal spermogram for *Pseudomonas* and *Prevotella*, respectively, vs. 52.7% of normal spermogram for *Lactobacillus*) [29]. More recently, in 2019, Baud et al. studying a European population, drew attention to the previously described bacterial communities, while finding an alteration of sperm parameters in the *Prevotella*-dominated compared to the *Lactobacillus*-dominated spermotype [30]. The latter was not significantly observed in the present study, which could be explained by the difference in targeted hypervariable regions.

Alpha diversity comparisons demonstrated very interesting and surprising results in the present cohort. At first, in samples with positive cultures, alpha diversity was found to be lower than in patients who did not demonstrate such a result. This intriguing information could be explained by different hypotheses, mainly supported by the conventional microbiological definition of this parameter. In this study, and in the usual clinical management of patients, a positive culture is defined as the identification in the culture of some specific (i.e., known to be implicated in pathogeny) bacteria at a significant threshold (depending on the species). This definition did not fully represent the possible diversity of non-pathogenic bacterial population, especially in hardly/non-culturable bacteria, which were easily detected by 16S DNA sequencing. Second, the spermatic parameter “morphology” was associated with differences in bacterial diversity, and morphology lower than 4% with restricted alpha diversity. Even if the predictive character of the fertility potential of a man remains debated for this parameter, an alteration of semen morphology indicates the presence of a lower functionality of the spermatozoa, especially in cases of monomorphic abnormality. Moreover, it seems important to justify this information to clinicians in order to adapt the management of couples [36]. Importantly, compared to the study by Baud et al., the present study did not observe a change in diversity in patients with abnormal sperm motility [30]. Finally, the difference among richness indices in the literature should be considered with regard to the difference of methodology, especially the targeted variable region (V1–V2 vs. V1–V3 in the present study) (Table 3) [29].

A difference in beta diversity was found regarding the spermatic parameter “count”, suggesting that there is greater dissimilarity in samples with abnormal counts. To the best of our knowledge, this observation is the first to demonstrate such a difference in spermatic microbiome, as Baud et al., using appropriate analyses, did not observe a difference in microbiota composition between specific defect subgroups [30]. That said, in studies that have focused on the vaginal microbiome, this restriction in alpha diversity has previously been proposed to be a predictive marker of in vitro fertilization outcomes, leading to higher rates of clinical pregnancy and live births [37,38]. This reflects the possibility that a microbiome biomarker determined in a specific niche (such as the vagina) could have completely discrepant properties in another niche, such as semen. Indeed, microbial diversity seems to be an ameliorative marker for sperm quality in men, whereas it could be considered to be a pejorative marker during embryo implantation and pregnancy in women.

The present study highlighted the modification of specific bacterial species. First, the enrichment of the *Prevotella* genus in samples from patients with negative sperm cultures was more frequent in the abnormal spermogram group. This is consistent with the study by Baud et al., who showed the same enrichment of *Prevotella* in the abnormal spermogram group [30] (Table 3). These results are consistent with those of Weng et al., who demonstrated that *Pseudomonas* or *Prevotella* predominance is associated with abnormal semen parameters. [29]. Similarly, Hou et al. demonstrated the negative association between *Anaerococcus* sp. and overall semen quality (i.e., volume, motility, and sperm count) [35]. More recently, Lundy et al., using 16S rRNA gene region V3-V4 index, suggested the same information about an inverse association with *Prevotella* and semen quality [31]. Contrary to data on diversity, this concordance on taxon presence could be justified due to the implication of this bacterial genus in vaginosis (considered to be female genital tract dysbiosis), which leads to a major impact on female fertility. Second, the enrichment of the *Haemophilus* genus can be observed in samples from patients with decreased sperm counts. Even if this observation has not been put forward in human studies, altered bovine sperm parameters (motility–vitality) were demonstrated in vitro in the presence of *Haemophilus* [39].

It bears mentioning that most of the methodologies used in the previously described studies focusing on sperm microbiota have been sequenced the hypervariable regions of the 16S rDNA V1 to V2 (Hou et al. and Baud et al. studies, [30,35]), V4 alone (Weng et al. [29]), or V3 to V4 [31] (Table 3). Herein, the modification of the discrimination capacities of the sequencing process (pyrosequencing vs. synthesis with reversible dye terminators), associated with amplification of the V1 to V3 regions, could explain some of the difference described therebefore, as is the case for alpha or beta diversities. Nevertheless, the observed differences are not mechanistic bonds and shall need to be fully analyzed in specific future analyses.

The results of the present study open up potential niches for probiotic therapeutics aimed at restoration, the objective being to improve sperm parameters and, consequently, male fertility. Up until now, studies aimed at developing therapeutic modification of the microbiome in fertility have been focused on the female partner. Studies dealing with the modulation of a potential endometrial microbiome in view of increasing the predominance of *Lactobacillus* have not yielded conclusive results on pregnancy rates and premature miscarriage [40]. For the male partner, one study demonstrated the effect of oral supplementation with *Lactobacillus* and *Bifidobacterium* (over a 6-week period) on sperm motility and DNA fragmentation, without exploration of the final benefit for live birth rate [41].

To conclude, this study calls for further in-depth studies of bacterial colonization of the male urogenital tract, with more extensive and multicentric recruitment. This type of study would yield greater extrapolation to the results obtained, paving the way to a more complete description of the spermatic microbiome and the definition of a bacterial signature correlated with predictable or observed spermatic abnormalities. Moreover, a longitudinal study of the sperm microbiome, and its modulation according to the time period of abstinence, could be of interest to determine whether variability in the sperm parameters, particularly the sperm count, could be associated with disturbances in the bacterial communities [42].

## Figures and Tables

**Table 2:** Comparison of beta diversities, determined with different estimated indices (Bray–Curtis and weighted UniFrac indices) according to sperm parameters (* *p* < 0.05).

		Bray–Curtis Index		Unifrac Weighted Index	
		Pseudo-F	*p*-Value	Pseudo-F	*p*-Value
**Sperm count (M of spermatozoa per ejaculation)**	≤39 vs. >39	3.05	0.022 *	3.71	0.025 *
**Motility (% of mobile spermatozoa)**	<32% vs. ≥32%	1.73	0.098	2.61	0.074
**Morphology (% of morphologically normal spermatozoa)**	<4% vs. ≥4%	0.80	0.508	0.59	0.585

**Table 3:** Main results of previous studies describing semen microbiota depending on semen biological parameters. CASA = computer-assisted semen analysis.

Studies	Weng et al., 2014 [29]	Baud et al., 2019 [30]	Hou et al., 2013 [35]
**Population**	36 normal semen parameters, 60 men with semen abnormalities	26 normal semen parameters, 68 men with semen abnormalities	19 healthy sperm donors, 58 men with semen abnormalities
**Technology**	MiSeq system (Illumina) Synthesis with reversible dye terminators	MiSeq system (Illumina) Synthesis with reversible dye terminators	Roche 454 GS-FLX (Roche-454 Life Sciences) Pyrosequencing
**Targeted sequences**	V4 16S rRNA gene	V1–V2 16S rRNA gene	V1–V2 16S rRNA gene
**Semen parameters**	Volume, mobility, count, morphology, anti-sperm antibody, leucocytes, and CASA	Count, mobility, and morphology	Volume, mobility, and count
**Most common species**	*Lactobacillus*, *Gardnerella*, *Pseudomonas*, *Prevotella*, *Streptococcus*, *Finegoldia*, *Haemophilus*, and *Rhodanobacter*	*Corynebacterium*, *Prevotella*, *Lactobacillus*, *Streptococcus*, *Staphylococcus*, *Finegoldia*, *Haemophilus*, and *Burkholderia*	*Ralstonia*, *Lactobacillus*, *Corynebacterium*, *Streptococcus*, *Prevotella*, *Finegoldia*, *Anaerococcus*, and *Peptinophilus*
**Groups**	*Pseudomonas* predominant (G1), *Lactobacillus* predominant (G2), *Prevotella* predominant (G3)	*Prevotella* enriched, *Lactobacillus* enriched, polymicrobial	6 groups characterized by high proportion of same species, but none predominant
**Associations**	G2 associated with normal semen parameters; G1 and G3 associated with abnormal semen parameters	*Prevotella* associated with defective semen mobility; *Lactobacillus* associated with normal semen morphology; *Staphylococcus* associated with normal semen parameters	No clusters were associated with semen parameters *Anaerococcus*: negative association with semen quality in general

## Data Availability

Raw data are available under the Sequence Read Archive BioProject PRJNA647447 (SRR12276169 to SRR12276257).
